# Evolving Blood Pressure Estimation: From Feature Analysis to Image-Based Deep Learning Models

**DOI:** 10.1007/s10916-025-02228-6

**Published:** 2025-07-09

**Authors:** Vishal Singh Roha, Rahul Ranjan, Mehmet Rasit Yuce

**Affiliations:** 1 Department of Electrical and Computer Systems, Monash University, Wellington Rd, Clayton, 3800 Melbourne, VIC Australia; 2 Faculty of Information Technology, Monash University, Wellington Rd, Clayton, 3800 Melbourne, VIC Australia

**Keywords:** Blood pressure, Cross-attention mechanism, Photoplethysmography, ResNet-50, Transfer learning

## Abstract

Traditional cuffless blood pressure (BP) estimation methods often require collecting physiological signals, such as electrocardiogram (ECG) and photoplethysmography (PPG), from two distinct body sites to compute metrics like pulse transit time (PTT) or pulse arrival time (PAT). While these metrics strongly correlate with BP, their reliance on multiple signal sources and susceptibility to noise from modern wearable devices present significant challenges. Addressing these limitations, we propose an innovative framework that requires only PPG signals from a single body site, leveraging advancements in artificial intelligence and computer vision. Our approach employs images of PPG signals, along with their first (vPPG) and second (aPPG) derivatives, for enhanced BP estimation. ResNet-50 is utilized to extract features and identify regions within the PPG, vPPG, and aPPG images that correlate strongly with BP. These features are further refined using multi-head cross-attention (MHCA) mechanism, enabling efficient information exchange across the modalities derived from ResNet-50 outputs, thereby improving estimation accuracy. The framework is validated on three distinct datasets, demonstrating superior performance compared to traditional PAT and PTT-based methods. Furthermore, it adheres to stringent medical standards, such as those defined by the Association for the Advancement of Medical Instrumentation (AAMI) and the British Hypertension Society (BHS), ensuring clinical reliability. By reducing the need for multiple signal sources and incorporating cutting-edge AI techniques, this framework represents a significant advancement in non-invasive BP monitoring, offering a more practical and accurate alternative to traditional methodologies.

## Introduction

Hypertension, commonly referred to as high blood pressure (BP), occurs when the force of blood against the artery walls remains persistently elevated, typically exceeding 140/90 mmHg. It is a major global health concern, affecting approximately 1.28 billion adults aged 30–79 years worldwide, as reported by the World Health Organization (WHO) in 2023 [1]. Among vital signs, BP plays a critical role in cardiovascular health, closely linked with heart rate, respiration rate, and heart rate variability (HRV). Studies have shown that HRV and metabolic markers influence cardiovascular risk, reinforcing the need for continuous monitoring [2]. Additionally, early detection of patient deterioration through real-time vital sign tracking has been explored in clinical settings, underscoring the importance of BP monitoring in assessing overall health [3]. Despite advancements, hypertension remains underdiagnosed, and machine learning (ML) has been investigated to enhance screening and risk prediction [4].

Traditionally, BP is measured using cuff-based devices. However, these methods are not really accurate for continuous or long-term monitoring due to their bulkiness and limited portability [5]. To address this, researchers have explored non-invasive BP estimation using bio-signals like electrocardiogram (ECG) and photoplethysmography (PPG). Pulse Arrival Time (PAT) and Pulse Transit Time (PTT), which reflect arterial stiffness and BP variations, have shown strong correlations with cardiovascular health [6, 7]. The rise of digital health has also led to the development of home BP monitoring (HBPM) and mHealth platforms, making standardized tracking more accessible [8]. However, real-time monitoring in critical care settings remains a challenge, with studies revealing that alarm thresholds are often static and fail to adapt to a patient’s condition, highlighting the need for more dynamic and intelligent monitoring systems [9].

Pulse Wave Velocity (PWV), derived from PTT, has also been widely studied for BP estimation [7]. [10] and [11] demonstrated significant correlations between PTT and BP, although these correlations vary among individuals [7, 12]. [12] proposed a chest-based continuous cuffless BP monitoring method using five different PAT estimations, highlighting the feasibility of using various physiological parameters for BP estimation. [13] introduced cuffless BP estimation algorithms utilizing PAT and other features such as heart rate and arterial stiffness index, achieving robust results for systolic BP (SBP) and diastolic BP (DBP) prediction. [14] extended this work by combining PPG morphological features with PAT, demonstrating their utility in large open databases.

Despite their potential, traditional feature-based methods face significant challenges. These approaches often rely on signals from two distinct body sites, such as ECG and PPG, to compute features like PAT, PTT, and PWV [7, 15]. This requirement increases system complexity and cost while being prone to errors from inter-signal synchronization and calibration issues [16]. Additionally, motion artifacts, signal variability, and inter-subject differences reduce the robustness and generalizability of these systems [12, 13]. While several works attempted regression models for BP estimation using PTT [17–19], they doesn’t demonstrate meeting the standard criteria. Oscillometry-based devices, commonly used in hospitals and homes, provide a single BP point without indicating the confidence interval of the measurement. [20] proposed an ensemble-based adaptive methodology to estimate confidence intervals for SBP and DBP, enhancing the reliability of oscillometric measurements.

To overcome these limitations, researchers have turned to deep learning (DL)-based methods that bypass manual feature extraction by directly modeling the relationship between raw PPG signals and BP. [21] introduced second-derivative PPG (SDPPG) features to enhance BP estimation, demonstrating improved accuracy by capturing vascular compliance information. Other works have integrated additional physiological parameters to refine BP prediction; [22] combined PPG signals with demographic data using two Gated Recurrent Unit (GRU) models to process dynamic patterns, while [23] proposed an unsupervised PPG representation learning framework (UPR-BP) that leverages unlabeled PPG data for accurate, non-invasive BP estimation. Beyond these, studies such as [24] and [25] have employed CNNs and transformer-based architectures, respectively, to enhance feature representation for BP estimation. The transformer-based approach in [25] captures high-dimensional dependencies in PPG signals, outperforming conventional models in real-time arterial BP monitoring.

Recent advancements have also explored image-based BP estimation, transforming PPG signals into visual representations for deep learning models. [26] introduced a BP estimation framework using five different PPG-derived image encodings, but its separate encoding process limits direct feature interaction, potentially missing cross-modal dependencies. [27] investigated image-based physiological signal processing, though it did not focus specifically on BP estimation. Similarly, [28] extended image-based BP estimation by incorporating PPG, and their derivatives (vPPG and aPPG) images, but each modality was processed independently, without leveraging their complementary information. Additionally, its reliance on Recursive Feature Elimination (RFE) with Random Forest for feature selection is computationally expensive and inefficient compared to end-to-end deep learning approaches. Other works, such as [29], have explored image-based transformations of PPG signals for enhanced feature extraction, demonstrating the potential of computer vision techniques in BP estimation.

Building on these advancements, we propose a cross-attention-based framework that effectively integrates information from PPG and its derivatives to enhance BP estimation. In our approach, PPG, vPPG, and aPPG are first transformed into visual representations, such as time-series plots, and passed through ResNet-50 [30] to extract robust embeddings. Unlike previous methods [28] that process these signals separately, we employ a multi-head cross-attention (MHCA) mechanism to facilitate information exchange between the embeddings, enabling the model to capture complementary patterns and improve estimation accuracy. This integrated approach addresses the limitations of earlier methods, demonstrating superior performance and robustness across diverse datasets.

**Algorithm 1:**
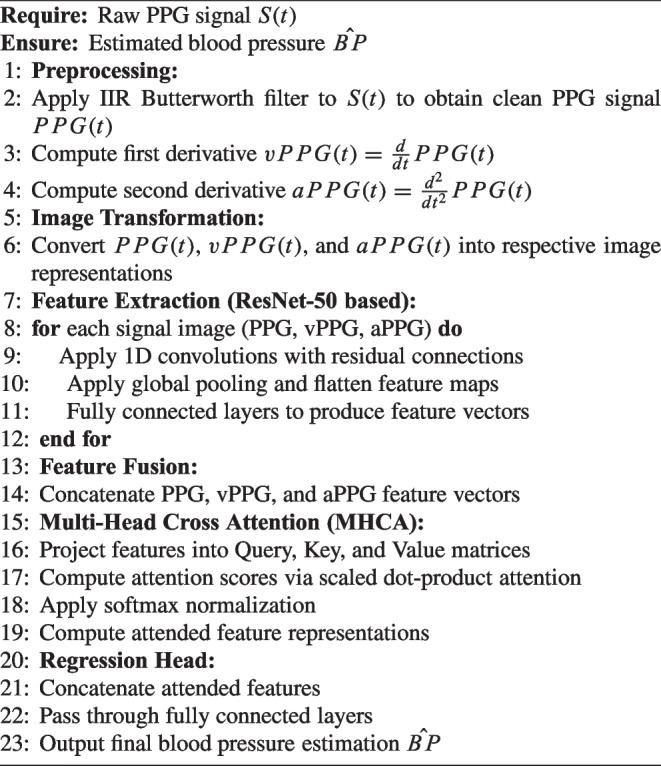
Pseudocode for the IMCA-PPG framework.

**Fig. 1:**
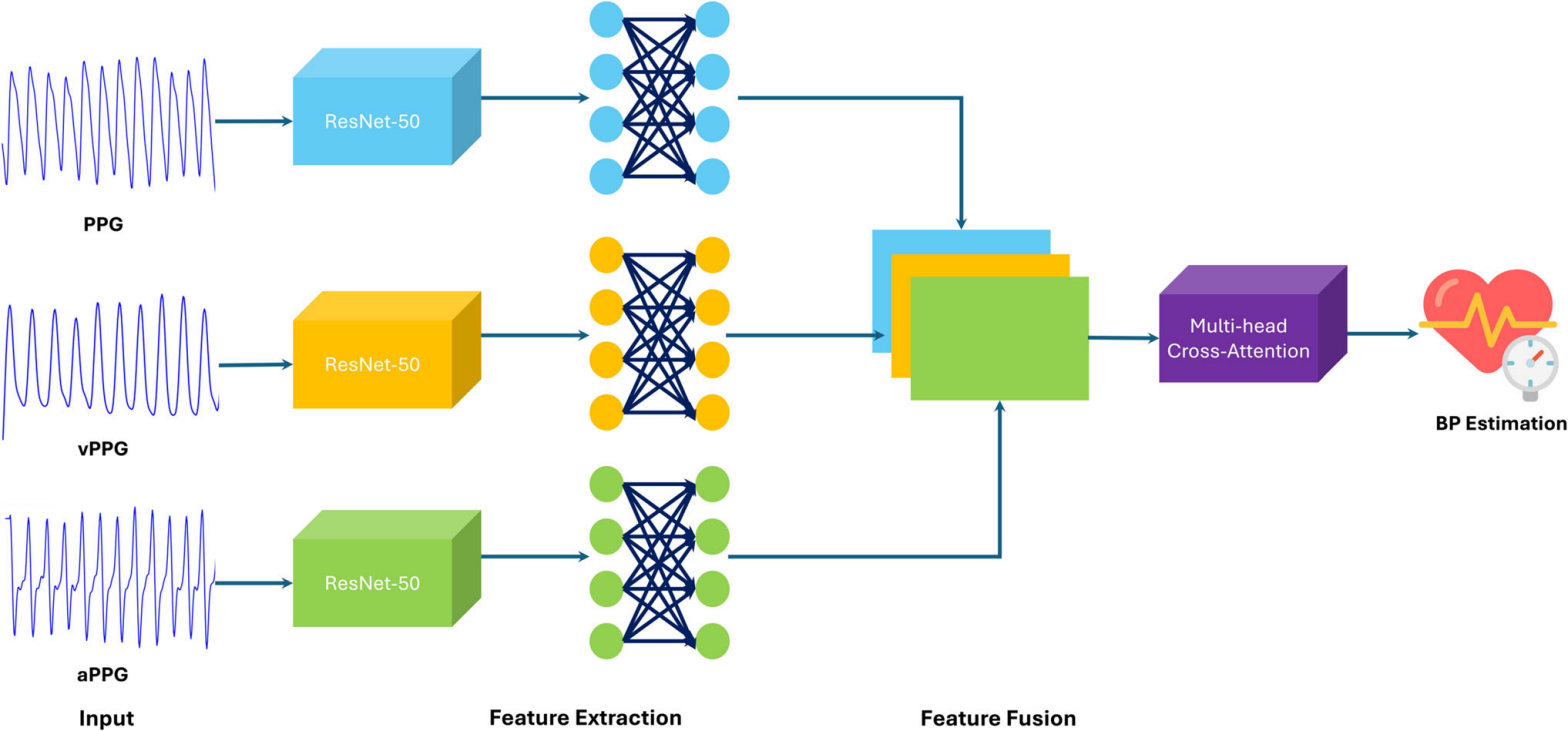
Image-Based Multimodal Cross-Attention Framework (IMCA-PPG) for BP estimation

Additionally, the framework minimizes reliance on extensive preprocessing, making it resilient to noise and motion artifacts commonly encountered in real-world data. By leveraging image-based representations and modern neural architectures, the proposed framework achieves a significant advancement in non-invasive, continuous BP monitoring. To validate its effectiveness, the framework is evaluated on two publicly available datasets, showcasing its robustness and generalizability. The primary contributions of this work are summarized as follows:

1. **Feature Extraction with ResNet-50:** Utilization of a pre-trained ResNet-50 model to extract robust feature embeddings from PPG, vPPG, and aPPG images, eliminating the need for manual feature engineering.
2. **MHCA mechanism for Fusion:** Utilization of a MHCA mechanism to fuse the extracted embeddings, enabling the model to capture interdependencies and improve BP estimation accuracy.
3. **Single-Site BP Estimation:** A convenient approach that estimates BP from a single PPG site, reducing hardware complexity and enhancing practicality.
4. **Robust Framework Evaluation:** Comprehensive validation on three diverse datasets to demonstrate the framework’s reliability and generalizability across populations and conditions.

The rest of the paper is structured as follows: “Methodology” section describes the Methodology and its components, including data utilized for assessment, feature extraction, MHCA mechanism and details the experimental setup such as, hyper-parameters, and evaluation metrics. “Results and Discussion” section presents the results analysis and there discussion, and “Conclusion & Future Perspectives” section concludes the work.

**Fig. 2:**
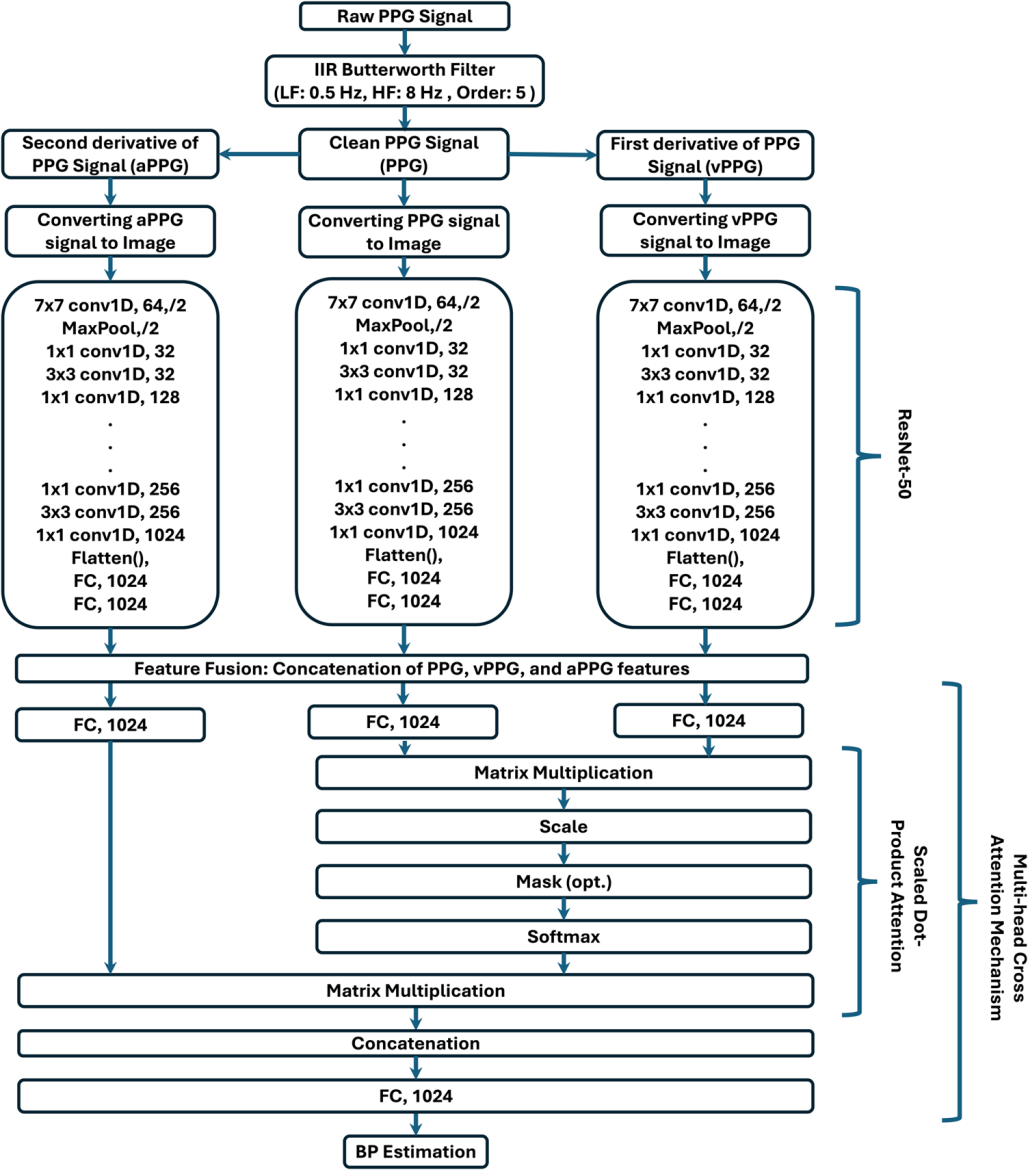
Flowchart of the proposed Image-Based MultiModal Cross-Attention Framework (IMCA-PPG) framework

## Methodology

This section provides a detailed description of the methodology underlying the proposed MultiModal Cross-Attention Framework (IMCA-PPG) framework for BP estimation (see Fig. 1). The IMCA-PPG framework is also summarized in Algorithm 1 which begins with signal preprocessing and transformation steps to derive multi-modal representations from raw PPG signals. The subsequent feature extraction phase leverages ResNet-50-based architectures to autonomously extract deep physiological features from PPG, vPPG, and aPPG modalities. These features are then fused using the proposed MHCA mechanism (see Fig. 2), designed to enhance inter-modality relationships and improve BP prediction accuracy. The section also outlines the datasets employed for validation, the hyperparameter configurations used for training, and the evaluation metrics adopted, ensuring a comprehensive understanding of the framework’s development and performance analysis.

**Fig. 3:**
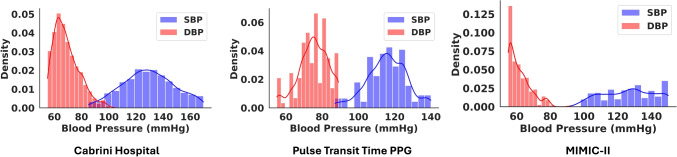
Distribution of SBP and DBP values across the three datasets. Each plot illustrates the density distribution of SBP and DBP measurements in mmHg, highlighting differences in participant populations across datasets

### Datasets Utilized for Framework Assessment

To evaluate the performance of our proposed framework, we utilized three datasets. The first dataset, referred to as the Pulse Transit Time PPG Dataset, is publicly accessible and consists of data collected from 22 subjects performing three physical activities: running, sitting, and walking [31]. This dataset served as the initial training set for developing the DL model. The second dataset, known as the Cabrini Hospital Dataset, was obtained through a collaboration with Cabrini Hospital in Melbourne, Australia, and includes data from 43 subjects, comprising both healthy individuals and those diagnosed with hypertension [12]. To complement these datasets and address the limitation of small sample sizes, we additionally utilized a subset of the MIMIC-II database [32], which contains physiological recordings from a large cohort of critical care patients. Specifically, we randomly selected data from 1000 participants to ensure a broader distribution of BP values and greater demographic variability compared to the other datasets. Detailed descriptions and relevant characteristics of these datasets are outlined below. Additionally, the distribution of SBP and DBP values across all the datasets is illustrated in Fig. 3, highlighting the variation in participant populations.

#### Pulse Transit Time PPG Dataset

This publicly accessible dataset includes recordings from 22 participants, comprising 7 females and 15 males, aged between 20 and 55 years, with an average age of 28.41 years. Participant heights range from 155 to 190 cm, and weights range from 45 to 100 kg. The dataset captures synchronized PPG and ECG signals, as well as SBP and DBP measurements. Data were collected during three physical activities—running, sitting, and walking—designed to simulate dynamic, real-world scenarios.

To ensure high temporal resolution, all sensors were connected to an ARM Cortex-M4 microcontroller running at 180 MHz, enabling data acquisition within a 2 ms window at a 500 Hz sampling rate. BP readings were obtained using an OMRON HEM-7322 monitor. The study received approval from the University of Sydney’s Human Research Ethics Committee (approval 2020/7059), and all participants provided written informed consent. For more detailed information on this dataset, refer to [31].

#### Cabrini Hospital Dataset

This dataset, collected in collaboration with Cabrini Hospital in Melbourne, Australia, includes data from 43 participants (23 males and 20 females) aged 30 to 72 years (mean age: 51.30 ± 11.30 years). The cohort comprises 27 healthy and 16 hypertensive individuals, providing a diverse range of physiological conditions. ECG and PPG signals were recorded at 1000 Hz, alongside BP measurements obtained using a cuffed sphygmomanometer and a Finapres finger cuff for beat-to-beat monitoring. The study was approved by the Cabrini Human Research Ethics Committee (CHREC170528) (07-19-06-17) and registered with the ANZ Clinical Trial Registry (ACTRN12617000774325p). All participants provided informed consent before inclusion in the study.

Data collection was structured into two phases. In the first phase, participants assumed three postures, each held for three minutes and repeated twice. In the second phase, participants were split into two groups: one group performed six exercise tasks (handgrip and cycling) to elevate BP, while the other group received Glyceryl Trinitrate (GTN) to lower BP under medical supervision. For both groups, left-ear PPG signals and beat-to-beat BP values were continuously recorded, providing comprehensive data for analysis. Additional details are available in Heydari et al. [12].

#### MIMIC-II Dataset

The MIMIC-II database is a publicly available resource comprising physiological signals and clinical data collected from critically ill patients admitted in Intensive Care Unit (ICU) [32, 33]. For this study, we extracted a subset of 1000 participants to address the limited number of subjects in the Pulse Transit Time PPG and Cabrini Hospital datasets. This subset includes continuous arterial BP (ABP) waveforms and PPG signals sampled at 125 Hz. Systolic and diastolic BP values were derived from the ABP waveforms by identifying the maximum and minimum pressure points for each cardiac cycle. The MIMIC-II cohort offers a broader range of BP values, as well as increased diversity in patient age and clinical conditions, thereby allowing for a more rigorous assessment of the framework’s generalizability. The inclusion of this larger and more heterogeneous dataset enhances the evaluation by providing greater coverage across different physiological and pathological states.

**Fig. 4:**
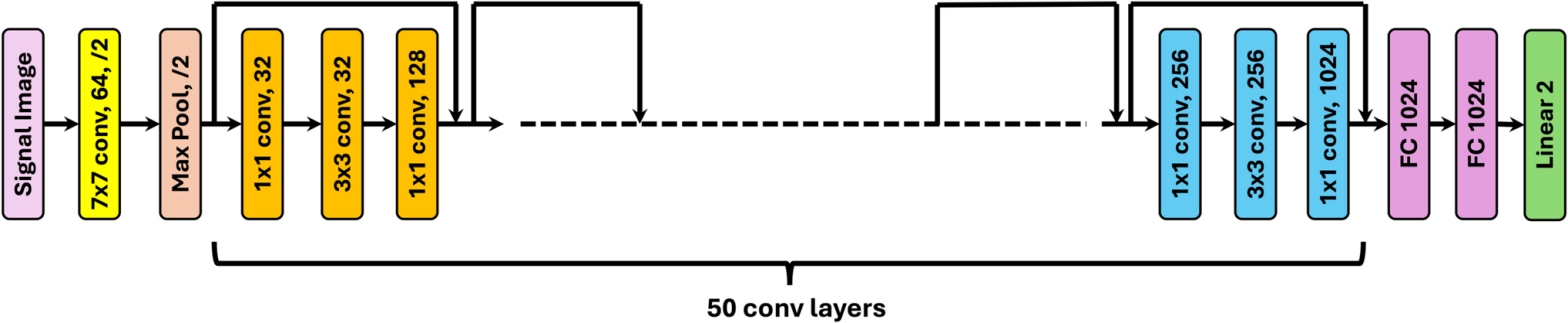
Modified ResNet-50 architecture for feature extraction from PPG, vPPG, and aPPG signal images

**Fig. 5:**
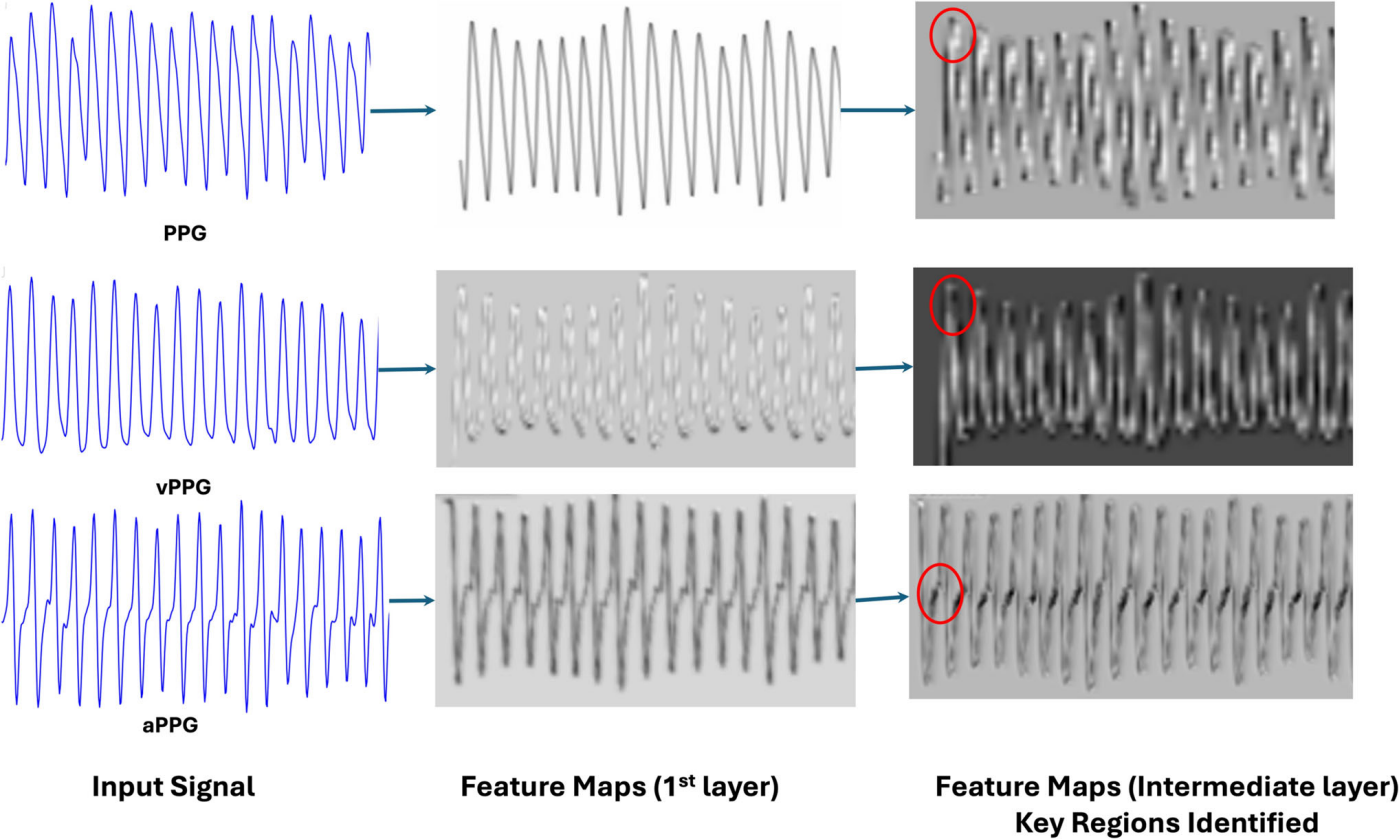
Key regions identified by ResNet-50 for BP estimation from PPG, vPPG, and aPPG images

### Signal Processing and Feature Extraction

The acquired PPG signals from the participants underwent rigorous preprocessing using the Neurokit2 toolbox [34]. Preprocessing began with signal cleaning through an Infinite Impulse Response (IIR) Butterworth filter using the SOS method. The filter parameters were precisely tuned with a lowcut frequency of 0.5 Hz, a highcut frequency of 8 Hz, and a filter order of 3. These parameters were chosen to preserve relevant signal components while minimizing noise, such as motion artifacts and baseline drift. Following this initial processing step, the vPPG and aPPG of the PPG signal were computed to uncover dynamic changes in the signals that are strongly indicative of BP variations.

To enable downstream analysis, the processed signals were segmented into 15-second overlapping windows with a sliding window of 66.67$\%$. Subsequently, each segment was plotted and saved as an image, capturing the distinct temporal patterns of PPG, vPPG, and aPPG. These images serve as an effective medium for leveraging advanced computer vision models, transforming raw signal data into a format amenable for DL-based feature extraction.

Feature extraction was carried out using a pre-trained ResNet-50 model [30], which has been widely recognized for its exceptional performance in extracting meaningful features from complex data (see Fig. 4). Trained on the $IMAGENET1K\_V1$ dataset [35], ResNet-50 brings several advantages to this application. Its use of residual connections effectively addresses vanishing gradient issues during training, enabling the model to capture deep hierarchical features. This capability is critical for identifying subtle yet critical regions of interest (ROIs) within the PPG, vPPG, and aPPG images. These ROIs are strongly correlated with BP variations, as illustrated in Fig. 5, demonstrating the model’s ability to identify physiologically meaningful patterns from the visualized signals.

**Fig. 6:**
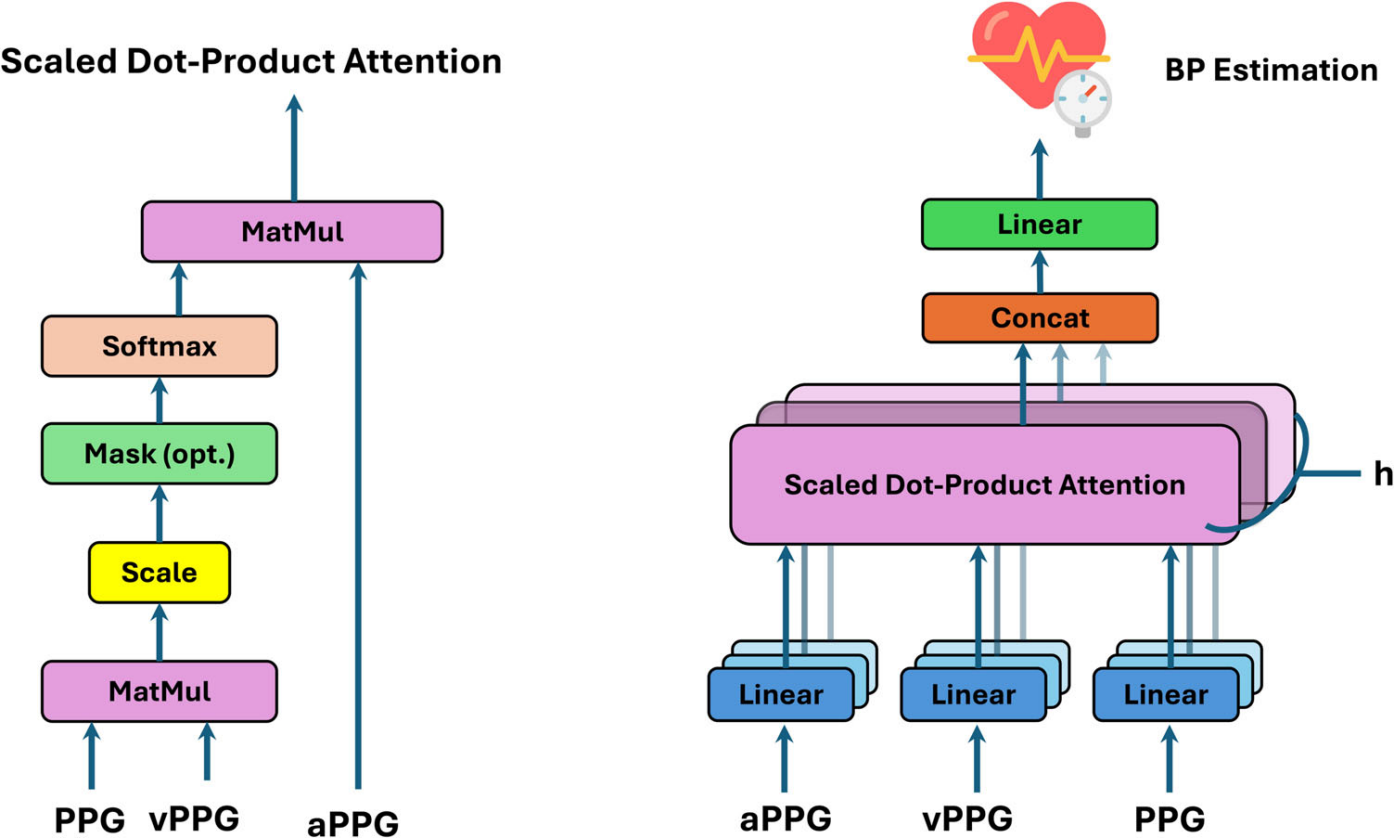
MHCA Mechanism for BP Estimation. The left diagram illustrates the scaled dot-product attention mechanism, while the right diagram represents the MHCA framework used in our approach. PPG serves as the query (Q), while vPPG and aPPG act as key (K) and value (V), enabling feature fusion for enhanced BP prediction

The ResNet-50 model was chosen over simpler architectures due to its ability to generalize effectively across different data modalities. Its deep convolutional layers allow for multi-level feature extraction, providing a richer understanding of signal characteristics that relate to BP. Furthermore, the pre-trained model significantly reduces the computational burden and training time, leveraging transfer learning to adapt well to the task of BP estimation.

### Multi-Head Cross-Attention Mechanism for Multi-Modal Feature Fusion

Extracting features from PPG, vPPG, and aPPG images using ResNet-50 provides a rich representation of physiological signals, but analyzing these modalities independently may overlook critical interdependencies. Since PPG captures volumetric blood flow, vPPG highlights the rate of change, and aPPG enhances pulsatility patterns, a framework that learns interactions across these modalities is essential for accurate BP estimation.

To address this, we employ a MHCA mechanism, inspired by the multi-head attention framework [36]. Unlike standard multi-head attention, which processes features within a single modality, MHCA explicitly learns cross-modal relationships, aligning PPG, vPPG, and aPPG to maximize predictive performance. As illustrated in Fig. 6, the proposed model utilizes PPG as the query (*Q*), vPPG as the key (*K*), and aPPG as the value (*V*), enabling a dynamic fusion of complementary features. The feature vectors extracted from PPG, vPPG, and aPPG using ResNet-50 are projected into query (*Q*), key (*K*), and value (*V*) spaces using learnable transformations:

1 $$\begin{aligned} Q_{PPG}= &   X_{PPG} W^Q, \quad K_{vPPG} = X_{vPPG} W^K, \nonumber \\ V_{aPPG}= &   X_{aPPG} W^V \end{aligned}$$

where $X_{PPG}$, $X_{vPPG}$, and $X_{aPPG}$ represent feature vectors from their respective modalities, and$W^Q$,$W^K$,$W^V$ are learnable weight matrices. The scaled dot-product attention, visualized in Fig. 6 (left), determines how strongly features from PPG (Q) align with vPPG (K) and aPPG (V):

2 $$\begin{aligned} \text {Att}(Q_{PPG}, K_{vPPG}, V_{aPPG}) = \text {softmax} \left( \frac{Q_{PPG} K_{vPPG}^T}{\sqrt{d_k}} \right) V_{aPPG} \end{aligned}$$

where $d_k$ is the dimensionality of the key vector, ensuring numerical stability. For MHCA, multiple attention heads operate in parallel, capturing diverse feature interactions across PPG, vPPG, and aPPG. The outputs of these heads are concatenated and projected into a unified space:

3 $$\begin{aligned} \text {MHCA}(Q_{\text {PPG}}, K_{\text {vPPG}}, V_{\text {aPPG}}) = \text {Concat}(\text {head}_1, ..., \text {head}_h) W^O \end{aligned}$$

where each attention ${head}_i$ is computed as:

4 $$\begin{aligned} \text {head}i = \text {Attention}(Q{\text {PPG}} W_i^Q, K_{\text {vPPG}} W_i^K, V_{\text {aPPG}} W_i^V) \end{aligned}$$

By aggregating multi-head attention outputs, we refine cross-modal feature interactions:

5 $$\begin{aligned} X_{\text {fusion}} = \frac{1}{h} \sum _{i=1}^{h} \text {MHCA}(Q_{\text {PPG}}, K_{\text {vPPG}}, V_{\text {aPPG}})_i \end{aligned}$$

where *h* represents the number of attention heads. Finally, the fused feature representation is mapped to BP predictions through a fully connected layer:

6 $$\begin{aligned} \hat{Y}_{BP} = W_{BP}\cdot X_{\text {fusion}} + b_{\text {BP}} \end{aligned}$$

where $W_{BP}$ and $b_{BP}$ are trainable parameters for SBP and DBP estimation.

### Hyperparameters & Evaluation metrics

The experiments utilized Batch Normalization and Max Pooling for regularization in ResNet-50, with convolutional layers (32, 64, 128, 256) and kernel sizes (1×1, 3×3), followed by Global Average Pooling. Both MHCA and ResNet-50 employed ReLU activation, the Adam optimizer (learning rate=0.0001), batch size=16, and 200 training epochs with a validation split of 0.1. To prevent overfitting and enhance generalization, K-fold validation (k=5), early stopping, and dynamic learning rate adjustment were implemented. These settings ensure robust generalization and high-performance BP estimation across diverse datasets.

In this study, we evaluate the effectiveness of our proposed framework using five distinct performance metrics: Coefficient of Determination ($R^2$), Root Mean Squared Error (RMSE), Mean Absolute Error (MAE), Mean Error (ME), and Standard Deviation (SD). To ensure statistical rigor and enhance interpretability, 95% confidence intervals were computed for all evaluation metrics and Bland–Altman agreement by utlizing the mean bias and limits of agreement (LoA) parameters with bootstrap resampling (1000 iterations, $\alpha$ = 0.05) [37]. Furthermore, the proposed approach is benchmarked against the AAMI [38] and BHS [39] standards, which are critical guidelines in the healthcare industry.

## Results and Discussion

Our proposed framework IMCA-PPG fundamentally shifts the paradigm by leveraging DL and MHCA fusion to extract richer feature representations from PPG signals. Through the transformation of PPG signals into structured image representations, combined with a state-of-the-art ResNet-50 [30] backbone, we demonstrate a superior approach for BP estimation. This section delves into the performance analysis, impact of MHCA fusion, dataset generalizability, compliance with clinical standards, and comparative analysis against existing literature, offering a holistic perspective on the efficacy of our approach.

**Table 1:** Comparison of SBP and DBP estimation accuracy across features and datasets, benchmarked against AAMI standards

Dataset	Features	SBP					DBP				
		$R^2$	RMSE	MAE	ME	SD	$R^2$	RMSE	MAE	ME	SD
PTT PPG	**PPG**	0.38 ± 0.04	10.78 ± 0.45	7.94 ± 0.31	0.10 ± 0.48	10.79 ± 0.45	0.43 ± 0.03	6.56 ± 0.23	5.01 ± 0.18	-0.22 ± 0.27	6.55 ± 0.21
	**vPPG**	0.93 ± 0.02	3.70 ± 0.57	1.17 ± 0.15	0.07 ± 0.16	3.70 ± 0.57	0.95 ± 0.01	1.98 ± 0.28	0.72 ± 0.08	0.09 ± 0.08	1.98 ± 0.29
	**aPPG**	0.40 ± 0.04	10.62 ± 0.44	7.79 ± 0.31	0.06 ± 0.47	10.62 ± 0.45	0.37 ± 0.04	6.89 ± 0.26	5.24 ± 0.18	0.14 ± 0.30	6.89 ± 0.26
	**MHCA**	**0.96** ± **0.00**	**2.80** ± **0.14**	**1.94** ± **0.08**	**-0.01** ± **0.12**	**2.80** ± **0.13**	**0.94** ± **0.01**	**2.14** ± **0.11**	**1.49** ± **0.06**	**0.11** ± **0.09**	**2.14** ± **0.11**
Cabrini	**PPG**	0.24 ± 0.07	13.69 ± 0.80	10.42 ± 0.59	-0.51 ± 0.58	13.69 ± 0.80	0.37 ± 0.06	7.33 ± 0.40	5.50 ± 0.32	0.58 ± 0.32	7.31 ± 0.42
	**vPPG**	0.30 ± 0.05	13.17 ± 0.65	10.28 ± 0.54	-0.55 ± 0.54	13.16 ± 0.70	0.38 ± 0.05	7.24 ± 0.40	5.50 ± 0.31	-0.01 ± 0.32	7.24 ± 0.41
	**aPPG**	0.16 ± 0.06	14.43 ± 0.71	11.42 ± 0.62	-1.41 ± 0.57	14.37 ± 0.67	0.30 ± 0.06	7.69 ± 0.41	5.80 ± 0.34	-0.26 ± 0.33	7.69 ± 0.43
	**MHCA**	**0.71** ± **0.02**	**8.15** ± **0.32**	**4.94** ± **0.20**	**0.01** ± **0.19**	**6.48** ± **0.25**	**0.64** ± **0.03**	**5.44** ± **0.22**	**3.59** ± **0.13**	**0.35** ± **0.13**	**4.09** ± **0.21**
MIMIC-II	**PPG**	0.28 ± 0.01	12.21 ± 0.19	9.83 ± 0.16	-1.22 ± 0.29	12.15 ± 0.19	0.04 ± 0.01	7.46 ± 0.19	5.82 ± 0.10	-0.65 ± 0.17	7.43 ± 0.19
	**vPPG**	0.86 ± 0.01	5.36 ± 0.24	2.87 ± 0.10	0.67 ± 0.12	5.32 ± 0.24	0.77 ± 0.02	3.63 ± 0.18	2.00 ± 0.07	0.39 ± 0.09	3.61 ± 0.17
	**aPPG**	0.94 ± 0.01	3.51 ± 0.22	1.26 ± 0.07	-0.11 ± 0.08	3.51 ± 0.22	0.89 ± 0.01	2.49 ± 0.17	0.96 ± 0.05	-0.24 ± 0.06	2.47 ± 0.19
	**MHCA**	**0.99** ± **0.00**	**1.13** ± **0.18**	**0.70** ± **0.05**	**-0.05** ± **0.04**	**0.82** ± **0.05**	**0.98** ± **0.00**	**1.09** ± **0.06**	**0.83** ± **0.03**	**0.04** ± **0.06**	**1.06** ± **0.06**
Medical Standard	**AAMI** [38]	**N/A**	**N/A**	**N/A**	$\le$ **5**	$\le$ **8**	**N/A**	**N/A**	**N/A**	$\le$ **5**	$\le$ **8**

Note: Results are reported as mean ± 95% confidence intervals obtained via bootstrap resampling

Bold entries are used to highlight the best-performing feature sets

**Table 2:** Comparison of proposed IMCA-PPG framework with other literature

Framework	Type of Approach	Dataset	Number of Subjects	Signals	Activities	SBP					DBP				
						$R^{2}$	RMSE	MAE	ME	SD	$R^{2}$	RMSE	MAE	ME	SD
Ahmad et al. [10]	Feature	Private	10	ECG	No (Sitting)	N/A	N/A	N/A	N/A	3.25	N/A	N/A	N/A	N/A	2.16
Nabeel et al. [40]	Feature	Private	35	PPG	No	0.55	6.33	N/A	1.15	7.98	0.59	6.33	N/A	0.86	6.36
Eom et al. [41]	Feature	Private	15	ECG, PPG, Ballistocardiogram	No	0.52	5.42	4.06	-0.20	4.04	0.49	4.30	3.33	-0.02	3.42
Byfield et al. [42]	Feature	Private	26	PPG	No	0.88	3.18	2.12	N/A	N/A	0.62	3.91	2.93	N/A	N/A
Ma et al. [23]	Feature	Aurora-BP	548	PPG	No	N/A	N/A	N/A	1.83	8.62	N/A	N/A	N/A	-0.69	5.31
Rizal et al. [22]	Feature	PPGBP	219	PPG	No	N/A	2.79	1.46	0.84	2.66	N/A	1.63	1.16	0.04	1.63
Lee et al. [25]	Feature	SCH BP Hospital	169	PPG	No	N/A	N/A	1.03	0.12	0.87	N/A	N/A	0.92	-0.64	0.79
Heydari et al. [12]	Feature	Cabrini Hospital	41	PPG, Bio-Impedance	Yes (Cycling, Handgrip)	0.88	N/A	N/A	N/A	-0.0005	0.92	N/A	N/A	N/A	N/A
Kachuee et al. [13]	Feature	MIMIC-II	942	ECG, PPG	No	0.35	N/A	11.17	-0.06	10.09	0.23	N/A	5.35	0.36	6.14
Kachuee et al. [13]	Feature	MIMIC-II	57	ECG, PPG	No	0.29	N/A	8.21	-0.06	5.45	0.32	N/A	4.31	0.36	3.52
Kachuee et al. [13]	Feature	MIMIC-II	57	ECG, PPG	No	0.29	N/A	8.21	-0.06	5.45	0.32	N/A	4.31	0.36	3.52
Esmaelpoor et al. [43]	Feature	MIMIC-II	200	PPG	No	0.90	N/A	N/A	1.91	5.55	0.90	N/A	N/A	0.67	2.84
Yang et al. [14]	Feature	MIMIC-III	1376	ECG,PPG	No	N/A	6.92	N/A	N/A	6.92	N/A	3.99	N/A	N/A	3.99
Yen et al. [44]	Feature	MIMIC-II	1551	ECG,PPG	No	N/A	N/A	2.24	0.02	3.59	N/A	N/A	1.40	-0.10	2.56
Zhang et. al. [24]	Feature	UCI	398	ECG,PPG	No	N/A	N/A	N/A	4.49	4.66	N/A	N/A	N/A	2.07	2.23
Sarkar et al. [45]	Feature	MIMIC-II	90	PPG	No	N/A	N/A	5.37	N/A	5.56	N/A	N/A	2.96	N/A	3.13
Ma et al. [23]	Feature	MIMIC-III	3070	PPG	No	N/A	N/A	N/A	0.30	4.68	N/A	N/A	N/A	0.25	2.66
Rizal et al. [22]	Feature	MIMIC-III	25	PPG	No	N/A	N/A	5.74	N/A	6.97	N/A	N/A	6.72	N/A	8.93
Lee et al. [25]	Feature	MIMIC-II	169	PPG	No	N/A	N/A	1.31	0.10	0.72	N/A	N/A	1.30	-0.42	0.89
Liu et al. (2023) [26]	Image	UCI (non-mixed)	450	PPG	No	N/A	13.03	9.19	N/A	N/A	N/A	5.05	3.81	N/A	N/A
Liu et al. (2023) [26]	Image	UCI (mixed)	450	PPG	No	N/A	4.62	3.06	N/A	N/A	N/A	2.35	1.61	N/A	N/A
Koparır et al. [28]	Image	MIMIC-II	942	vPPG	No	0.67	N/A	5.63	0.13	8.83	0.46	N/A	2.82	0.52	8.79
Our Work (IMCA-PPG)	Image	MIMIC-II	1000	PPG, vPPG, aPPG	No	**0.99**	**1.13**	**0.70**	**-0.05**	**0.82**	**0.98**	**1.09**	**0.83**	**0.04**	**1.06**
Our Work (IMCA-PPG)	Image	Cabrini Hospital	43	PPG, vPPG, aPPG	Yes (Postures, Cycling, Handgrip)	**0.71**	**8.15**	**4.94**	**0.01**	**6.48**	**0.64**	**5.44**	**3.59**	**0.35**	**4.09**
Our Work (IMCA-PPG)	Image	PTT PGG	22	PPG, vPPG, aPPG	Yes (Sit, Run, Walk)	**0.96**	**2.80**	**1.94**	**-0.01**	**2.80**	**0.94**	**2.14**	**1.49**	**0.11**	**2.14**

Bold entries indicate our proposed framework’s results

**Table 3:** Evaluation of SBP and DBP estimation compliance with BHS standards

Dataset/	Features	SBP			DBP		
Medical Standard		$\le 5$ mmHg	$\le 10$ mmHg	$\le 15$ mmHg	$\le 5$ mmHg	$\le 10$ mmHg	$\le 15$ mmHg
PTT PPG	**PPG**	42.49	72.47	86.78	59.63	87.58	96.98
	**vPPG**	95.14	97.36	98.54	96.74	99.06	99.67
	**aPPG**	43.67	73.23	86.59	59.02	86.36	96.36
	**MHCA**	**91.08**	**99.34**	**99.95**	**96.08**	**99.67**	**99.95**
Cabrini	**PPG**	34.72	58.26	73.96	57.19	83.47	94.41
	**vPPG**	32.94	57.43	75.62	56.48	83.83	95.24
	**aPPG**	27.23	52.56	71.11	54.93	82.40	94.17
	**MHCA**	**71.57**	**83.92**	**90.77**	**78.33**	**93.03**	**97.34**
MIMIC-II	**PPG**	31.06	57.24	77.75	47.36	88.30	96.72
	**vPPG**	86.84	92.70	96.44	91.05	96.88	98.94
	**aPPG**	91.98	96.75	98.74	94.94	98.61	99.54
	**MHCA**	**99.35**	**99.93**	**100.00**	**99.93**	**100.00**	**100.00**
BHS [39]	**Grade A**	**60**	**85**	**95**	**60**	**85**	**95**
	**Grade B**	**50**	**75**	**90**	**50**	**75**	**90**
	**Grade C**	**40**	**65**	**85**	**40**	**65**	**85**

Bold entries are used to highlight the best-performing feature sets

A critical aspect of our evaluation is the validation across three heterogeneous datasets—PTT PPG [31], Cabrini [12], and MIMIC-II [32]—each presenting distinct challenges. The PTT PPG dataset, collected under controlled conditions, ensures high signal fidelity, whereas the Cabrini dataset introduces real-world variability through changes in posture, movement artifacts, and externally induced BP fluctuations. On the other hand, MIMIC-II dataset, comprising ICU-admitted patients, exhibits smoother PPG waveforms due to reduced peripheral resistance and diminished diastolic features, as evident from the SBP and DBP distributions in Fig. 3, making BP estimation more challenging. Despite these variations, our framework consistently maintains high performance across datasets, as shown in Table 1. To ensure unbiased evaluation, ResNet-50 was trained separately for each dataset, preventing cross-dataset feature leakage and ensuring that reported metrics reflect dataset-specific learning.

The integration of MHCA further enhances BP estimation by capturing interdependencies among PPG, vPPG, and aPPG images. Unlike conventional feature-based methods, MHCA adaptively emphasizes critical physiological regions within each modality, notably the diastolic peak and notch, which are strongly correlated with BP variations. As presented in Table 1, individual modalities perform suboptimally when analyzed separately. However, MHCA fusion enables the framework to extract complementary patterns from all three signal representations, outperforming PPG, vPPG, and aPPG across multiple evaluation metrics.

For the PTT PPG dataset, MHCA improves upon PPG by 152.63%, 74.02%, and 75.57% in $R^2$, RMSE, and MAE for SBP, respectively. Compared to aPPG, MHCA achieves gains of 140.00%, 73.63%, and 75.09%. While vPPG attains lower RMSE and MAE for SBP, MHCA consistently provides the best overall performance by achieving the highest $R^2$ and balanced accuracy across both SBP and DBP estimation. For DBP, MHCA offers improvements of 118.60%, 67.99%, and 70.46% over PPG, and 154.05%, 69.52%, and 71.76% over aPPG in $R^2$, RMSE, and MAE, respectively. On the Cabrini dataset, the gains are even more substantial: MHCA improves over PPG by 195.83%, 40.52%, and 52.61% for SBP in $R^2$, RMSE, and MAE, respectively, and by 64.86%, 26.05%, and 34.73% for DBP. For the MIMIC-II dataset, despite the smoother PPG morphology and lower BP variability, MHCA improves over PPG by 253.57%, 90.67%, and 92.88% for SBP, and by 216.28%, 70.75%, and 85.75% for DBP in $R^2$, RMSE, and MAE, respectively. Notably, vPPG outperforms PPG and aPPG on PTT PPG and Cabrini datasets, where BP variability is externally induced through exercise and posture changes, emphasizing systolic and diastolic fluctuations captured effectively by the first derivative. Conversely, for MIMIC-II, aPPG shows improved performance by accentuating subtle changes in the diastolic notch and peak in smoother PPG waveforms, aligning with observations in Koparır et al. [28], where aPPG achieved the best performance using ResNet-50 across PPG derivatives. All results are reported as mean ± 95% confidence intervals obtained via bootstrap resampling in Table 1, ensuring statistical rigor and reliability.

**Fig. 7:**
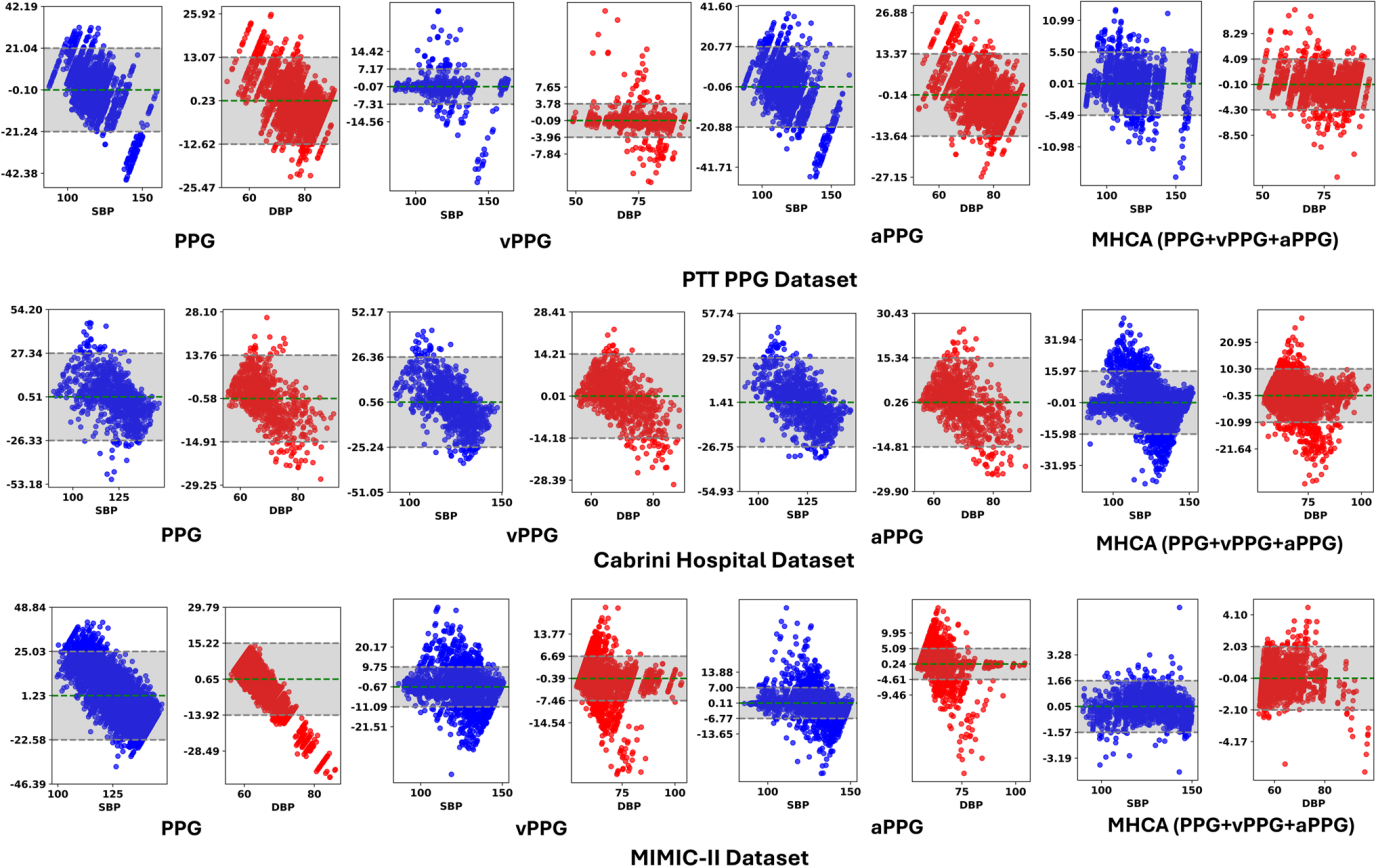
Bland-Altman plots illustrating the agreement between estimated and reference SBP and DBP values across different feature representations (PPG, vPPG, aPPG, and MHCA) across all datasets. The green dashed line represents the mean bias, while the grey dashed lines indicate the upper and lower LoAs. Shaded areas represent the 95% confidence intervals for the mean bias and the LoA (grey)

These results not only establish the effectiveness of MHCA but also align with findings from Koparır et al. [28], where vPPG outperformed PPG and aPPG. However, while Koparır et al. [28] evaluated their model on the MIMIC-II [32] dataset with limited BP variability due to ICU patient data, our framework demonstrates robust generalization across datasets with externally induced BP fluctuations (e.g., exercise and posture variations). Compared to Koparır et al. [28], our IMCA-PPG model achieves a 47.76% higher $R^2$ and lower MAE of 87.57% for SBP estimation on MIMIC-II, showcasing substantial improvements in both accuracy and reliability. Furthermore, Heydari et al. [12] proposed a chest-based cuffless BP monitoring system leveraging five distinct PAT definitions derived from PPG and bio-impedance signals under controlled physical activity. While effective, their approach requires additional bio-impedance hardware and multiple signal channels, complicating real-time deployment and increasing hardware dependencies. In contrast, our IMCA-PPG framework operates solely on single-site PPG measurements and generalizes across different datasets and acquisition settings, making it inherently hardware-agnostic and more suitable for scalable, real-world healthcare applications (Table 2).

For any BP monitoring framework to be clinically viable, compliance with established medical standards is essential. As demonstrated in Tables 1 and 3, our IMCA-PPG framework satisfies the stringent AAMI [38] and BHS [39] standards across all datasets. For the PTT PPG dataset, the model achieves a ME of -0.01 ± 0.12 mmHg for SBP and 0.11 ± 0.09 mmHg for DBP, both well within the AAMI $\le$5 mmHg requirement. The corresponding SD values are 2.80 ± 0.13 mmHg for SBP and 2.14 ± 0.11 mmHg for DBP, comfortably meeting the $\le$8 mmHg threshold. On the Cabrini dataset, ME values of 0.01 ± 0.24 mmHg for SBP and 0.35 ± 0.12 mmHg for DBP, and SD values of 6.48 ± 0.25 mmHg and 4.09 ± 0.21 mmHg, respectively, further confirm compliance. In addition, for the larger and clinically diverse MIMIC-II cohort, IMCA-PPG achieves ME values of -0.05 ± 0.04 mmHg for SBP and 0.04 ± 0.06 mmHg for DBP, with corresponding SDs of 0.82 ± 0.05 mmHg and 1.06 ± 0.06 mmHg, significantly surpassing AAMI thresholds. Evaluation under BHS standards demonstrates that our model consistently achieves Grade ‘A’ classifications. For the PTT PPG dataset, 91.08% of SBP and 96.08% of DBP predictions fall within 5 mmHg, far exceeding the 60% requirement for Grade ‘A’. Similarly, the Cabrini dataset attains 71.57% and 78.33% within 5 mmHg for SBP and DBP, respectively, achieving Grade ‘B’ for SBP and Grade ‘A’ for DBP. On the MIMIC-II dataset, IMCA-PPG achieves an outstanding 99.35% and 99.93% within 5 mmHg for SBP and DBP, respectively, securing a perfect Grade ‘A’ for both measurements.

These results highlight the clinical reliability of our method, making it a strong candidate for integration into practical healthcare applications. By addressing real-world challenges and ensuring generalizability across diverse physiological conditions, IMCA-PPG advances the state of cuffless BP estimation, paving the way for its adoption in everyday health monitoring.

A comparative analysis with prior studies further underscores the advantages of our framework. While conventional approaches have employed various machine learning and feature-based techniques, our model consistently outperforms these methods, as evidenced in Table 2. Prior work often relies on multi-sensor setups, such as ECG and PPG combinations [13, 14, 44] or PPG on two different sites, which increase hardware complexity. In contrast, our single-site PPG-based model outperforms feature-based methods and recent image-based frameworks in terms of $R^2$, RMSE, and MAE in terms of SBP and DBP estimations on the MIMIC-II dataset, while maintaining a streamlined and hardware-independent configuration. The ability to maintain high accuracy while simplifying hardware requirements represents a major step forward in non-invasive BP monitoring.

**Fig. 8:**
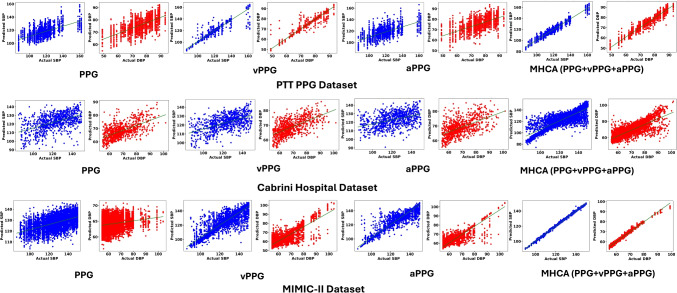
Linear fit plots showing the correlation between estimated and reference BP values, highlighting predictive accuracy across feature representations (PPG, vPPG, aPPG, and MHCA) across all datasets

Deep Learning models are often criticized for their black-box nature; however, our framework provides interpretability through ResNet-50 feature visualization. Figure 5 illustrates the key regions identified in PPG, vPPG, and aPPG images that contribute to BP estimation. These feature maps reveal that the model focuses on physiologically relevant variations, aligning with known BP-related signal characteristics. The automated extraction of these features eliminates the need for manual engineering, demonstrating the capability of DL to autonomously learn meaningful representations that enhance predictive accuracy.

To further validate our framework, we conducted Bland–Altman [46] and linear fit analyses. The Bland–Altman plots (Fig. 7) illustrate minimal bias and narrow LoA, confirming that most prediction errors lie within the 95% confidence bounds. The cumulative error distributions reinforce that the majority of SBP and DBP predictions fall within clinically acceptable thresholds. Similarly, the linear fit analysis (Fig. 8) exhibits strong correlation patterns, with high $R^2$ values, affirming the strong predictive capability of our model across heterogeneous datasets and modalities. These quantitative and visual analyses jointly substantiate the clinical robustness and generalizability of IMCA-PPG.

To assess the computational efficiency of the framework, all experiments were conducted using an NVIDIA A40 GPU with 48 GB VRAM and PyTorch 2.0.1 with CUDA acceleration. The ResNet-50 models used for feature extraction from PPG, vPPG, and aPPG representations each have an on-disk size of 102.8 MB, while the MHCA module introduces an additional 21 MB. On average, the training time for a single ResNet-50 model is approximately 52 minutes (3120 seconds), with an average inference time of 5192 milliseconds per sample. For the complete MHCA module, the average inference time per sample is 30.85 milliseconds, with a total training time of approximately 105 minutes (6300 seconds).

Although the current configuration delivers state-of-the-art performance, further reductions in model size and computational overhead are achievable through post-training optimizations such as pruning and quantization. These techniques preserve predictive accuracy while significantly lowering memory and compute requirements, enabling real-time deployment even in resource-constrained environments such as mobile applications and web servers. Based on existing benchmarks, models compressed to under 50,MB can achieve inference latencies below 30,ms and memory usage under 10,MB on standard CPUs and mobile processors [47]. While our current implementation uses PyTorch, models can be exported to ONNX format to ensure cross-platform compatibility across Android, iOS, Windows, Linux, macOS, and embedded systems. Further compression with ONNX Runtime quantization [48] enhances deployment feasibility for clinical or wearable applications.

Beyond computational efficiency, the proposed framework addresses key limitations of conventional BP monitoring methods. Traditional cuff-based devices provide only intermittent measurements and may cause discomfort during long-term use, particularly in ambulatory or home settings. In contrast, our framework leverages a single-sensor PPG signal, simplifying data acquisition and reducing hardware complexity. Unlike multi-sensor approaches that require synchronized ECG and PPG signals [13, 14, 24, 44], our system enables scalable, low-cost deployment without compromising accuracy. The incorporation of multiple PPG signal derivatives—PPG, vPPG, and aPPG—allows the extraction of complementary physiological features linked to arterial compliance, vascular aging, and peripheral resistance [49]. Feature maps generated by the ResNet-50 models, as shown in Fig. 5, consistently emphasize key waveform regions such as the systolic peak and diastolic notch. These regions are clinically recognized markers of cardiovascular health and arterial stiffness, enhancing the physiological interpretability and trustworthiness of the model’s predictions.

Transitioning from raw waveform-based to image-based BP estimation presents significant advantages. Traditional feature engineering approaches demand extensive domain expertise and signal preprocessing, limiting scalability and adaptability. In contrast, the deep learning-based framework developed in this study autonomously extracts clinically meaningful features, ensuring robustness across diverse sensor configurations. By combining single-site PPG acquisition with multi-modal image representations—PPG, vPPG, and aPPG—the system enhances estimation accuracy without increasing hardware complexity, paving the way for scalable, real-time BP monitoring solutions suitable for integration into commercial wearable devices and smartphone applications. With advancements in smartphone technology—specifically improved camera quality, higher resolution, and increased frame rates—the acquisition of remote PPG signals has become more reliable, enabling accurate, contactless BP estimation. Given its lightweight architecture and robust performance, the proposed framework can be seamlessly adapted for mobile or web-based platforms, facilitating continuous, real-time BP monitoring in non-clinical environments. Such deployment would support preventive healthcare initiatives and chronic disease management. Future work will involve implementing and validating the framework in these real-world settings to assess usability, accuracy, and clinical applicability in real-world settings.

## Conclusion & Future Perspectives

Our results establish the effectiveness of DL-based BP estimation, demonstrating that MHCA fusion significantly enhances predictive accuracy. By leveraging PPG, vPPG, and aPPG images through ResNet-50 feature extraction, our framework outperforms existing methodologies while adhering to established medical standards. The validation across three diverse datasets highlights its generalizability, confirming its suitability for real-world applications. Moving forward, we aim to expand this research by applying our framework to smartphone-based PPG data, enabling real-time, non-contact BP monitoring. This advancement holds immense promise in democratizing BP assessment, offering a scalable and accessible solution for continuous cardiovascular health monitoring. By bridging the gap between DL innovations and real-world usability, our approach lays the groundwork for future developments in AI-driven, non-invasive healthcare solutions.

## Data Availability

The data used in this study includes both publicly available and private datasets. The publicly available datasets, “Pulse Transit Time PPG Dataset” by Mehrgardt et al. (PhysioNet 10, 215–220, 2022), can be accessed at https://physionet.org/content/pulse-transit-time-ppg/1.1.0/ and “MIMIC-II” can be accessed at https://archive.ics.uci.edu/dataset/340/cuff+less+blood+pressure+estimation. The private dataset used in this study is not publicly available due to confidentiality restrictions. Further details regarding the data are provided within the manuscript.

## Code Availability

The code used in this study is not publicly available but can be made available upon reasonable request, subject to approval from the corresponding authors.
